# Performance of ChatGPT in Solving Questions From the Progress Test (Brazilian National Medical Exam): A Potential Artificial Intelligence Tool in Medical Practice

**DOI:** 10.7759/cureus.64924

**Published:** 2024-07-19

**Authors:** Mateus Rodrigues Alessi, Heitor A Gomes, Matheus Lopes de Castro, Cristina Terumy Okamoto

**Affiliations:** 1 School of Medicine, Universidade Positivo, Curitiba, BRA; 2 Neonatology, Universidade Positivo, Curitiba, BRA

**Keywords:** academic performance/grades, biomedical technology, medical codes of ethics, examination questions, expert systems, artificial intelligence and education, artificial intelligence in medicine

## Abstract

Background

The use of artificial intelligence (AI) is not a recent phenomenon, but the latest advancements in this technology are making a significant impact across various fields of human knowledge. In medicine, this trend is no different, although it has developed at a slower pace. ChatGPT is an example of an AI-based algorithm capable of answering questions, interpreting phrases, and synthesizing complex information, potentially aiding and even replacing humans in various areas of social interest. Some studies have compared its performance in solving medical knowledge exams with medical students and professionals to verify AI accuracy. This study aimed to measure the performance of ChatGPT in answering questions from the Progress Test from 2021 to 2023.

Methodology

An observational study was conducted in which questions from the 2021 Progress Test and the regional tests (Southern Institutional Pedagogical Support Center II) of 2022 and 2023 were presented to ChatGPT 3.5. The results obtained were compared with the scores of first- to sixth-year medical students from over 120 Brazilian universities. All questions were presented sequentially, without any modification to their structure. After each question was presented, the platform’s history was cleared, and the site was restarted.

Results

The platform achieved an average accuracy rate in 2021, 2022, and 2023 of 69.7%, 68.3%, and 67.2%, respectively, surpassing students from all medical years in the three tests evaluated, reinforcing findings in the current literature. The subject with the best score for the AI was Public Health, with a mean grade of 77.8%.

Conclusions

ChatGPT demonstrated the ability to answer medical questions with higher accuracy than humans, including students from the last year of medical school.

## Introduction

ChatGPT is an artificial intelligence (AI) tool that gained notable popularity in 2023 for its ability to generate texts, synthesize complex information, answer questions, and interpret phrases within seconds [1]. Unlike previous AI tools, which used models to learn patterns, this new technology developed by OpenAI operates under Large Language Models through deep learning. In general terms, this means that the tool is trained to predict the probability of a given word sequence based on its context from an immense database, spread across several layers of complexity, and it can learn from its own mistakes. Therefore, as more information is fed into ChatGPT, it becomes capable of solving problems and generating sentences it has never encountered before [2].

A recent article that gained significant attention in the academic community was published by Kung et al. in 2023, demonstrating that ChatGPT could pass the United States Medical Licensing Examination (USMLE) [2]. The exam, divided into three phases with over 1,000 questions, covers foundational medical knowledge to diagnostic and treatment skills. Unlike the United States, Brazil does not have a single national exam used to select future residents, with each hospital or hospital group conducting its own exam. Therefore, this article aimed to apply the tool’s knowledge to solve questions from the Progress Test (PT), a national medical exam administered at most Brazilian universities for first to sixth-year medical students. This test aims to evaluate discrepancies in teaching quality between universities, identify potential weaknesses to be corrected by the end of the sixth year, provide self-assessment for students, and train them for future residency exams.

The PT is conducted annually and contains 120 questions, with 20 questions related to Basic Sciences, Pediatrics, Internal Medicine, Surgery, Gynecology and Obstetrics, and Public Health. Its recent editions have involved over 50,000 students from 120 universities. In 2021, the Brazilian Association of Medical Education (ABEM) conducted a single national test, while in 2022 and 2023, the tests were divided by Brazilian regions [3]. These two recent evaluations have regional reach, covering certain clusters of states in the country. One of these clusters is the Southern Institutional Pedagogical Support Center II (Napisul II), comprising 13 universities from Paraná and Santa Catarina. Researchers accessed the questions and answer keys from this regional test and included them in the study. It is worth noting that this test is conducted by the same group, with the same number of questions, covering the same six topics. The difficulty and the evaluation method of the questions are similar.

Over the past decades, significant advancements in neural networks and AI have led to notable improvements in various fields, ranging from manufacturing and finance to consumer products. However, despite the advancements seen in these industries, the ability to apply this model to clinical care remains limited [4]. In this context, the present study aims to evaluate ChatGPT’s capacity for clinical reasoning by testing its performance on PT questions.

## Materials and methods

The study utilized an observational, cross-sectional design to evaluate the performance of ChatGPT 3.5 on 333 questions from the 2021 National Progress Test and the Regional Tests (Napisul II) of 2022 and 2023, excluding questions with images, nullified questions, and repeated questions. Each question was manually inputted into ChatGPT 3.5, and the platform’s history was cleared and restarted after each question to avoid memory bias. Responses were categorized as correct, incorrect, or insisted answers, with discrepancies reviewed by three blinded professors from Positivo University. ChatGPT’s performance was compared to the average scores of first to sixth-year medical students using appropriate statistical methods to assess accuracy and effectiveness.

Inclusion and exclusion criteria

Questions from the 2021 National Progress Test and the Regional Tests (Napisul II) of 2022 and 2023, totaling 360 questions (120 from each test) were included. We excluded questions that included images or figures containing graphs, questions nullified by the test organizers, and questions that repeated among the three tests evaluated.

As a result, 333 multiple-choice questions (MCQs) were used, with 109 from the 2021 PT, 117 from the 2022 Napisul II Test, and 107 from the 2023 Napisul II Test, each containing a single best-choice MCQ among five possible alternatives (A, B, C, D, or E).

Procedures

Initially, each question within the inclusion criteria was given to the ChatGPT 3.5 platform, and then categorized into the following three possible answers based on the official answer sheet of each test: correct answer, incorrect answer, considered more than one answer as correct. To avoid memory bias by the AI, the platform’s history was deleted, and the site was restarted after each question.

For incorrect answers, the phrase the correct answer is the letter “X,” where “X” contained the correct answer’s letter (e.g., A, B, C, D, or E), was appended to the response to allow the platform to agree or disagree with the proposed answer key.

Every question where ChatGPT disagreed with the official answer sheet was presented to three professors from Positivo University, Curitiba, Brazil, corresponding to each test area, namely, Basic Sciences, Public Health, Internal Medicine, Surgery, Pediatrics, and Gynecology and Obstetrics. All participating professors were sent an informed consent form, which had to be completed for participation in the study.

Professors then indicated whether they agreed with ChatGPT. Questions where two or more professors agreed with the AI were considered to have the official answer sheet wrong and labeled as “Insistence with validation in favor of ChatGPT.” If two or more professors agreed with the official answer key, it was labeled as “Insistence with validation of the official answer sheet.” It is important to note that the professors answered the questions at the same time, independently, and without knowing the correct answer according to the official sheet or the AI.

In cases where the platform considered more than one answer correct, the question “Which is the most correct alternative?” was asked to obtain a single alternative and improve statistical interpretation. If the new AI response matched the official answer key, it was considered correct; otherwise, it was considered incorrect.

Thus, the following four possible results were obtained for each question posed to ChatGPT: (A) Correct answer: correct responses on the first inquiry. (B) Incorrect answer: responses where the platform disagreed with the answer key but agreed after exposure. (C) Insistence with validation in favor of ChatGPT: after the platform disagreed with the answer key and insisted on a different answer, the professors’ evaluation favored ChatGPT’s response. (D) Insistence with validation of the official answer key: after the platform disagreed with the answer key and insisted on a different answer, the professors’ evaluation favored the official answer key.

The results were compared with the medical students’ scores, divided into the average score (average scores of students from first to sixth year) and only the sixth-year students. The results were also divided by the six major test areas. Unfortunately, for the 2021 test, the average percentage of correct answers by subject for students from the first to sixth year was not made available by ABEM, with only the percentage by subject for sixth-year medical students publicly provided. As the data was provided only in averages, a significance test could not be conducted.

## Results

**Figure 1:**
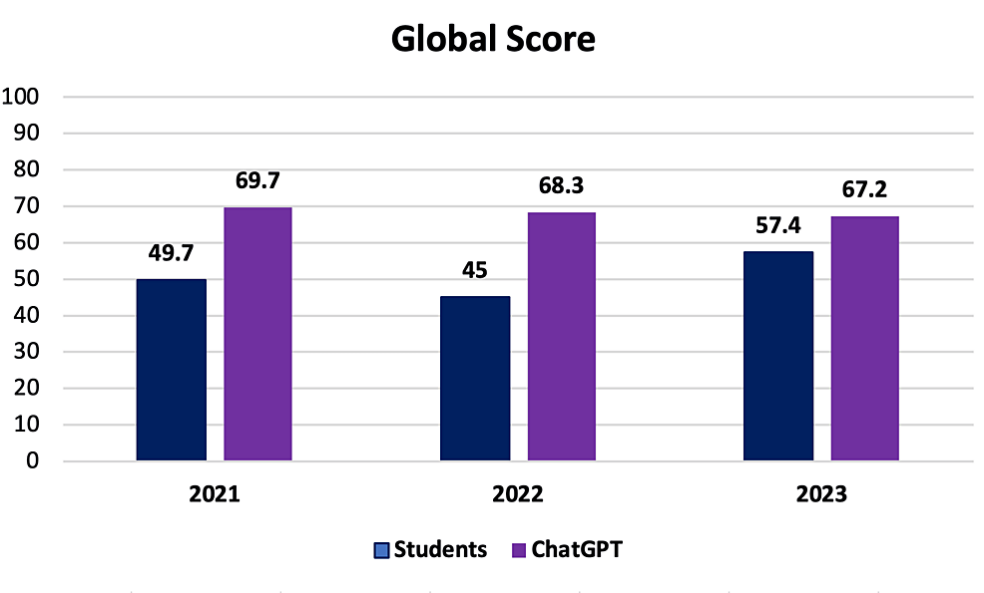
Score by year. Percentage score by each year of all students and ChatGPT.

Based on the questions that met the inclusion criteria, ChatGPT achieved average correct rates of 69.7%, 68.3%, and 67.2% for the 2021, 2022, and 2023 tests, respectively. In comparison, the students who took these same tests had average scores of 49.7%, 45.2%, and 57.4% in each evaluated year, as shown in Figure 1.

**Figure 2:**
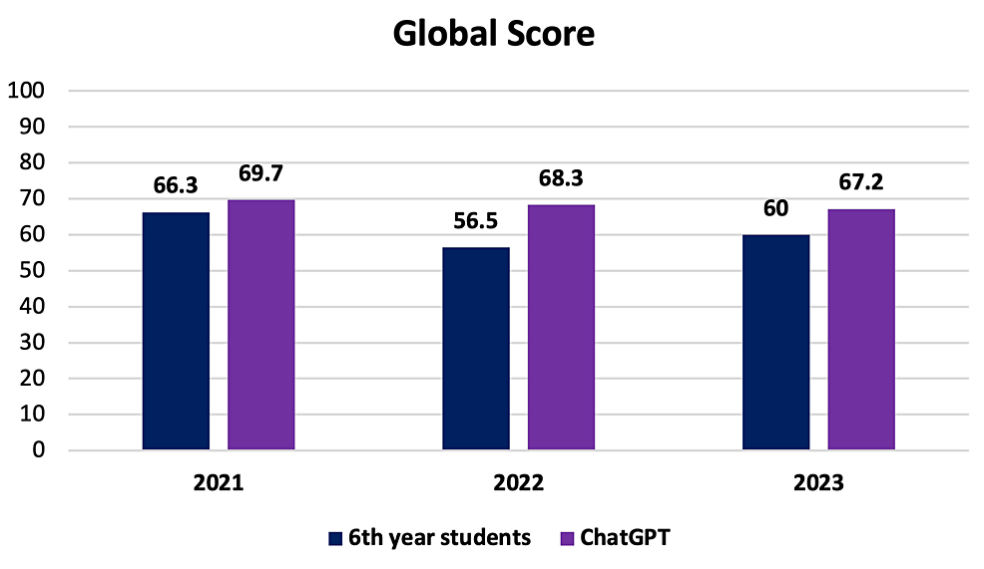
Score by year of sixth-year students. Percentage score by each year of only sixth-year students and ChatGPT.

When analyzing only sixth-year medical students, their correct rates on the three tests were 66.3%, 56.5%, and 60%, respectively, reaffirming the AI platform’s superiority in the average number of correct answers, as depicted in Figure 2.

**Figure 3:**
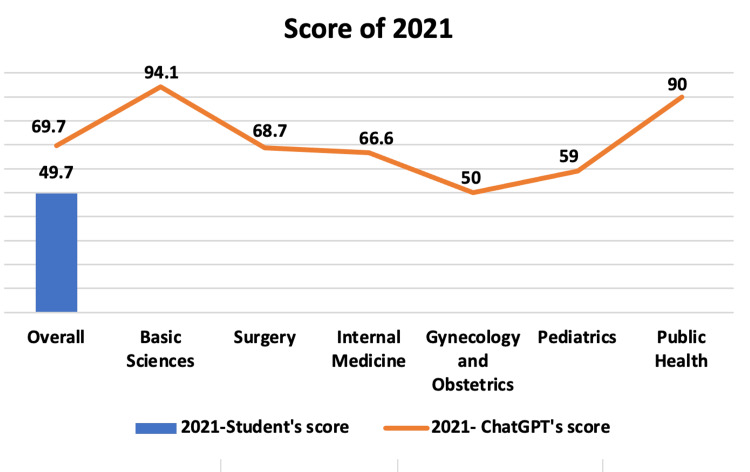
Score of 2021. Subject percentage score of all students and ChatGPT in 2021. There are no student scores for each subject because this data was not provided by the Brazilian Association of Medical Education.

For the 2021 exam, due to the unavailability of student performance data by subject, as previously mentioned, it was not possible to compare students’ scores by subject with those of ChatGPT. However, when comparing overall scores, the results were 49.7% for students and 69.7% for ChatGPT. Additionally, it is worth noting the platform’s accuracy by subject to visualize its performance by major area and compare it with tests from other years. The scores found were 94.1% (Basic Sciences), 68.7% (Surgery), 66.6% (Internal Medicine), 50% (Gynecology and Obstetrics), 59% (Pediatrics), and 90% (Public Health), as presented in Figure 3.

**Figure 4:**
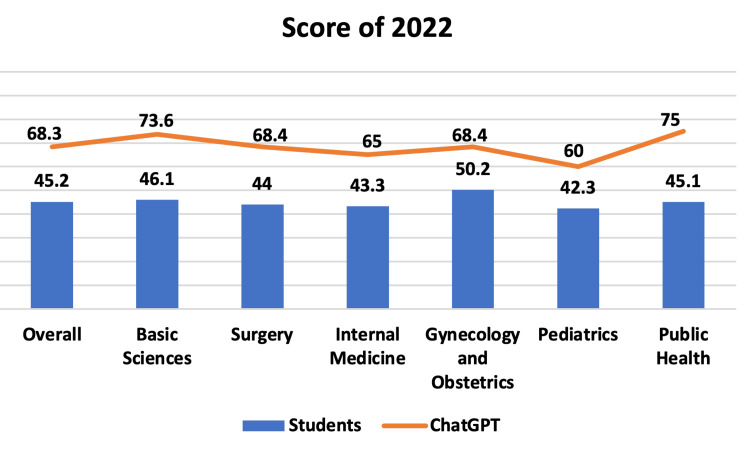
Score of 2022. Subject percentage score of all students and ChatGPT in 2022.

Comparing the accuracy of students who took the exam versus ChatGPT, it was found that in 2022, the overall score was 45.2% for students and 68.3% for the AI. When evaluating the average accuracy by subject of students and AI, the results were, respectively, 46% vs. 73.6% (Basic Sciences); 44% vs. 68.4% (Surgery); 43.3% vs. 65% (Internal Medicine); 50.2% vs. 68.4% (Gynecology and Obstetrics); 42.3% vs. 60% (Pediatrics); and 45.1% vs. 75% (Public Health), as illustrated graphically in Figure 4.

**Figure 5:**
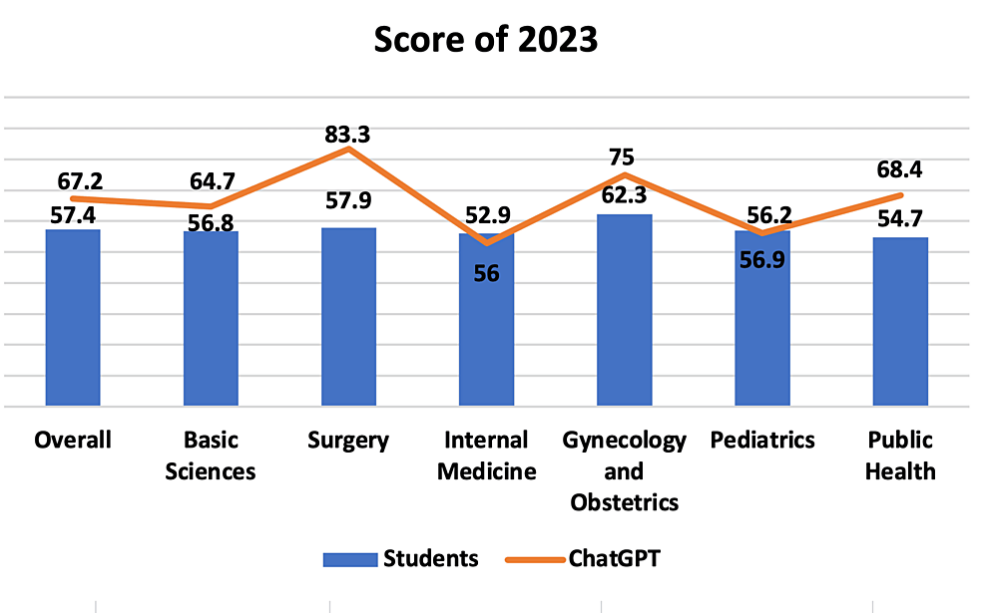
Score of 2023. Subject percentage score of all students and ChatGPT in 2023.

In 2023, the overall score for students was 57.4% compared to 67.2% for the platform. By specific subject, the values for participants and ChatGPT were, respectively, 56.8% vs. 64.7% (Basic Sciences); 57.9% vs. 83.3% (Surgery); 56% vs. 52.9% (Internal Medicine); 62.3% vs. 75% (Gynecology and Obstetrics); 56.9% vs. 56.2% (Pediatrics); and 54.7% vs. 68.4% (Public Health), as illustrated graphically in Figure 5.

**Figure 6:**
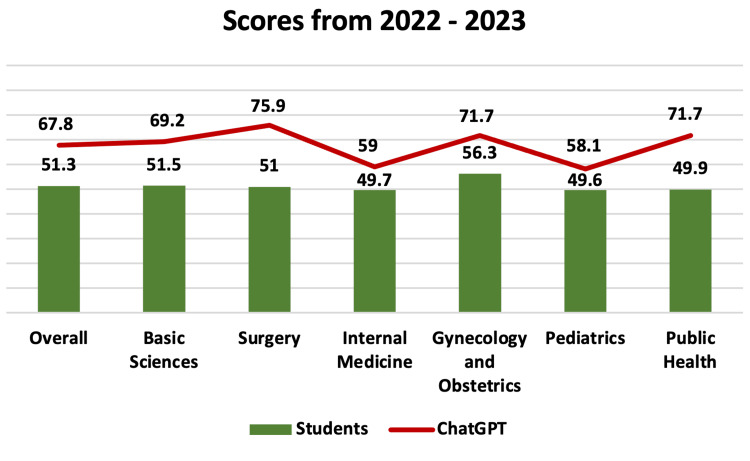
Score from 2022 to 2023. Average percentage score of 2022 plus 2023 tests of all students and ChatGPT, according to each subject.

Summing the overall average accuracy in the three tests, we noted an accuracy of 50.76% for students and 68.4% for ChatGPT. To compare the average performance by subject of all students, the results of the 2022 and 2023 tests were evaluated (2021 was not evaluated due to data unavailability). Thus, the performance of students vs. ChatGPT was observed to be, respectively, 51.5% vs. 69.2% (Basic Sciences); 51% vs. 75.9% (Surgery); 49.7% vs. 59% (Internal Medicine); 56.3% vs. 71.7% (Gynecology and Obstetrics); 49.6% vs. 58.1% (Pediatrics); and 49.9% vs. 71.7% (Public Health), as presented in Figure 6.

**Figure 7:**
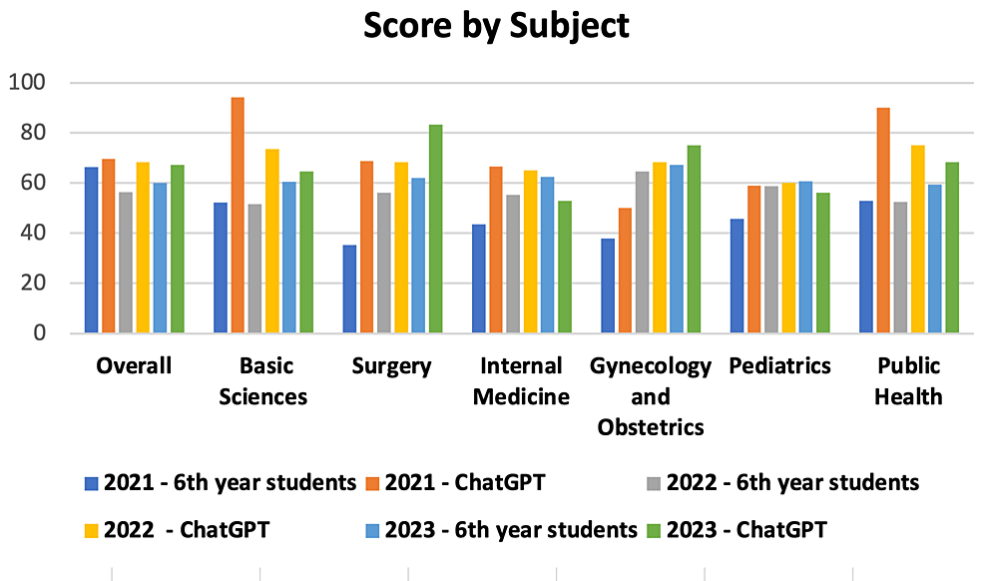
Score by subject. Score in percentage between students and ChatGPT and the respective year of the test, grouped according to subjects.

Evaluating the overall and subject-specific accuracy of sixth-year students in 2021, 2022, and 2023, the results were, respectively, 66.3%, 56.5%, and 60% for overall accuracy; 52.2%, 51.6%, and 60.5% for Basic Sciences; 35.3%, 56.1%, and 62.1% for Surgery; 43.5%, 55.4%, and 62.5% for Internal Medicine; 38%, 64.7%, and 67.2% for Gynecology and Obstetrics; 45.8%, 58.7%, and 60.7% for Pediatrics; and, finally, 53%, 52.5%, and 59.4% for Public Health. Conversely, the AI results for the same years and subjects were, respectively, 69.7%, 68.3%, and 67.2% for overall accuracy; 94.1%, 73.6%, and 64.7% for Basic Sciences; 68.7%, 68.4%, and 83.3% for Surgery; 66.6%, 65%, and 52.9% for Internal Medicine; 50%, 68.4%, and 75% for Gynecology and Obstetrics; 59%, 60%, and 56.2% for Pediatrics; and 90%, 75%, and 68.4% for Public Health. These results are graphically illustrated in Figure 7.

**Figure 8:**
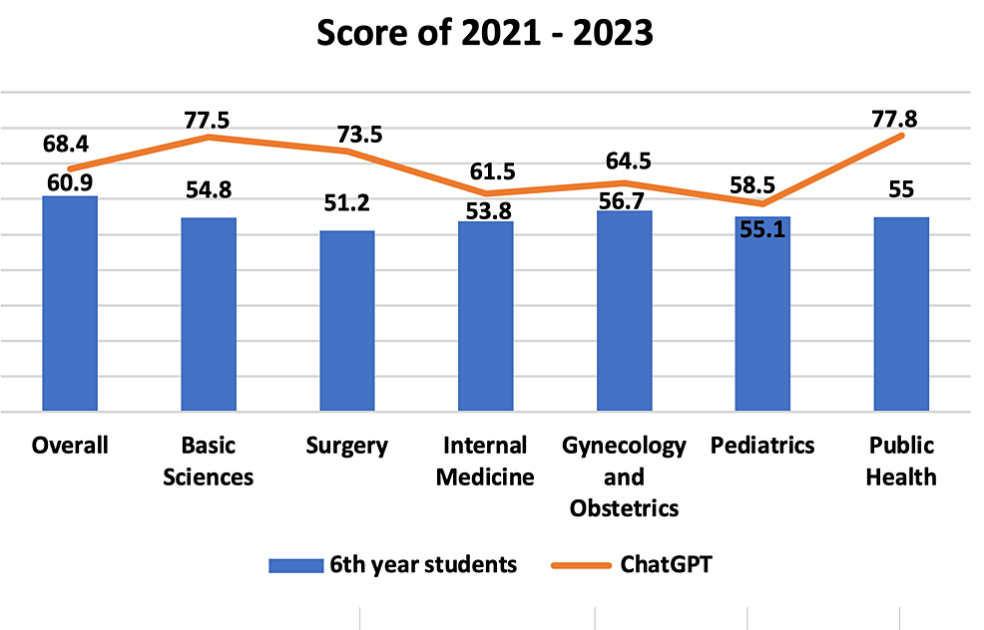
Score of 2021 to 2023. Average percentage score of 2021, 2022, and 2023 of sixth-year students and ChatGPT according to each subject.

With these numbers, it is possible to compare the overall and specific subject accuracy of sixth-year students vs. ChatGPT in the years 2021, 2022, and 2023, obtaining the following results, respectively: 60.9% vs. 68.4% (Overall), 54.8% vs. 77.5% (Basic Sciences), 51.2% vs. 73.5% (Surgery), 53.8% vs. 61.5% (Internal Medicine), 56.7% vs. 64.5% (Gynecology and Obstetrics), 55.1% vs. 58.5% (Pediatrics), and 55% vs. 77.8% (Public Health), as illustrated in Figure 8.

**Figure 9:**
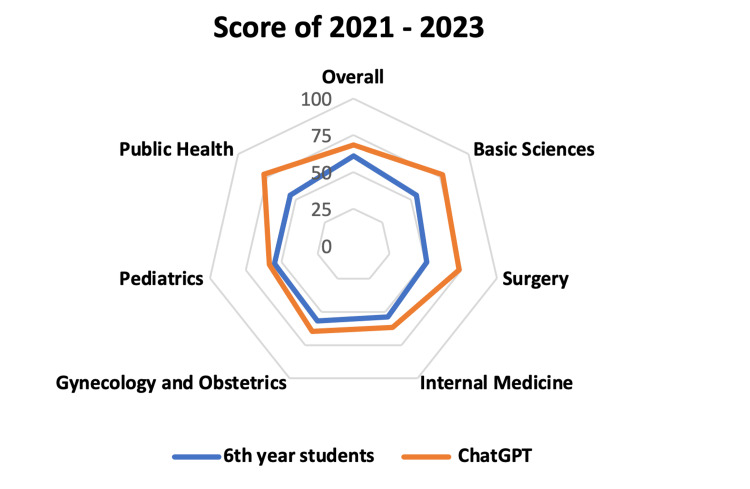
Radar graph: score of 2021 to 2023. Average percentage score of 2021, 2022, and 2023 of sixth-year students and ChatGPT, according to each subject. In the radar graphic, the inner lines represent a lower score, whereas the outside ones a better score.

In descending order from the highest to the lowest score, and averaging the scores over the three years, the AI showed greater accuracy in Public Health, Basic Sciences, Surgery, Gynecology and Obstetrics, and Internal Medicine, followed by Pediatrics, as can be seen in Figure 9.

In nine questions, ChatGPT disagreed with the official answer sheet and insisted on another answer, with only one of them having two or more professors agreeing with the AI platform, indicating another alternative as correct instead of the official answer.

## Discussion

AI has been the subject of many studies, especially after the popularization of ChatGPT, but there have been publications evaluating its application in Medicine since 2004 [5]. Its main advantages include creativity and speed, allowing its use in research, diagnosis, monitoring, planning, and medical education [6]. The accuracy in answering medical questions in the study was high and superior to the students, reinforcing how powerful this tool is, given that it is not connected to the internet to obtain information, as its last data source update was in 2021 [7-10]. The ability to generate original texts or based on the provided information is so complex that it can surpass tools that evaluate plagiarism or if the sequence of words was generated by humans. Its use in the preparation of scientific articles is already a topic of debate, and there are already works that have included AI as a co-author, something not recommended by major scientific journals [11-13].

Several concerns and ethical dilemmas have emerged after the beginning of research using ChatGPT and its applications in medicine. The main criticisms and dangers include the ability to create false and incorrect information, biased answers, the difficulty in distinguishing reliable sources from unreliable ones, and errors in citing information sources [11,14,15]. Additionally, the fields of ethics and patient confidentiality, as well as copyright laws, are still in a gray area, without laws that fully define their scope of action [11]. The way users utilize AI information can also make them complicit in criminal activities [16]. As already mentioned, the platform is not connected to the internet and searches for its answers from the information presented to it up to 2021, which in itself creates a significant bias given the speed of updates in science [14].

Comparing the performance of AI in answering USMLE questions with the PT, both showed a success rate close to or above 60% [2]. Evaluating the American study, one can observe an increase in ChatGPT’s accuracy in each tested USMLE exam (higher in Step 3 and lower in Step 1), considering there is a common perception among students that the difficulty decreases with each exam (Step 1, Step 2, and Step 3). As the Brazilian tests used in this study tend to maintain a similar difficulty pattern, the fact that the platform obtained close results in each test reaffirms the tendency that its performance has a direct relationship with the complexity of the questioning, as well as being able to maintain similar results when confronted with technically similar questions.

In Brazilian exams, its accuracy was even higher, surpassing students from the first to the sixth year. All three exams showed a higher accuracy rate in favor of AI. The main speculated reasons for a higher success rate in the PT are easier question levels, topics involving less information discovered after 2021, use of questions that require memorized knowledge instead of logical reasoning, the way the questions were elaborated, and the learning acquired by ChatGPT over time between the two studies. This is another study indicating the high ability to correctly answer medical questions, constituting the beginning of a database that will assist in validating AI for health-related topics [17].

When analyzing this study with Gobira et al. (2023), which applied ChatGPT 4.0 to answer the 2022 Brazilian National Examination for Medical Degree Revalidation (Revalida), a test that permits foreign physicians to work in Brazil, the number of correct answers was much higher in the study by Gobira et al., with an 87.7% global score [18]. In contrast to the present study, the worst score of the AI in the Revalida test was Public Health, while in PT it was the best subject score. Nevertheless, when examining the scores of this same subject, AI scored 77.8% in this study and 81.3% in the study by Gobira et al., demonstrating that their worse score was higher than ours. Although the revalidation test is considered a more difficult exam than PT, two hypotheses may explain the difference found. The first one is that ChatGPT 4.0 was used instead of 3.5 and the second is that the questions were clearer and less ambiguous, as Revalida is a more serious and important test.

Given the nature of medical knowledge exams and the way their questions are formulated, a question arises about the quality of medical education assessment. It is a fact that memorizing a large number of information is part of building a theoretical framework that allows the development of clinical reasoning. However, what is the validity of investing time and brain storage space to memorize meticulous concepts, knowing we cannot win this battle against AI? [4]

It is questioned whether it would be wiser to assume a symbiosis between doctor and machine to provide better healthcare, from both a technical and human perspective. To this end, both qualities, of equal value, would be exalted to their maximum potential, as both parties would be focused on their original designations. We are in a transition period, where it is valid to question whether the current tools for medical evaluation and education are indeed the most ideal, or if we are prioritizing memorization over logical reasoning and soft skills [19].

Endorsing the idea of linking medical education and practice to the use of AI, and similar to what was presented in the articles by Kung et al. (2023) and Gilson et al. (2024), the present study showed that ChatGPT’s responses were highly coherent [2,17]. In this way, a medical student could easily follow the platform’s internal language and logic to study multiple medical subjects, for example. Its internal insight and low level of contradiction reinforce that the AI presents consistent clinical reasoning, as well as high-quality explanations. Moreover, AI is capable of bringing up new and non-obvious insights that may not occur to students, generating novel concepts that can enhance human reasoning and learning [2,17].

Today it is already possible to affirm that this new form of technology will not only be part of human work but will replace it in various areas. Most scientists have already stipulated probable dates when AI will be able to perform certain activities better than or equal to humans, and many of these activities involve health services [20]. Every day new articles present the use of machine learning in image interpretation, clinical reasoning, analysis of laboratory tests, use in drug development, and much more [21-25]. Throughout evolution, humans have demonstrated a remarkable capacity for adapting to changes. Fighting against progress is a lost battle, as those who refuse to keep up with technological development inevitably fall behind and are replaced. It can be speculated that in the coming years, individual qualities will change drastically. While in the past, characteristics that most resembled machines were the most desired (memory, calculation, productivity, resilience, and obedience) by society, in the future, what will be more valuable is what makes us more human: empathy, creativity, abstraction, leadership, communication, intuition, and flexibility. These characteristics will be essential to navigate and thrive in an increasingly automated and technologically advanced world.

The study shows the strength of ChatGPT 3.5 in answering medical questions with higher accuracy than medical students, showcasing AI’s potential in medical education and assessment. The comprehensive evaluation across multiple years and subjects underscores the robustness of these findings. Several limitations need consideration such as the use of average student scores from ABEM limits the ability to perform detailed statistical significance analyses. The AI’s last data update was in 2021 and, currently, the platform is at version 4.0, while at the time of the present study, it was at version 3.5, which could affect its performance in more recent analysis. Additionally, the optional nature of the PT introduces potential selection bias, as not all students or universities participate. The validation process by professors, while necessary, could also introduce subjective bias. Addressing these limitations in future studies, such as using more recent AI versions and individual student data, could provide even more insightful results.

## Conclusions

ChatGPT demonstrated the ability to answer medical questions with higher accuracy than humans, including students from the last year of medical school. Its skills indicate its excellent knowledge of how medicine is studied in Brazil and the United States. More than just answering questions, it is creating debates and reflections in various areas of society, especially when it presents results similar to those of humans, prompting the reflection on whether we are truly inferior or merely self-evaluating incorrectly.
